# Intraoperative Assessment of Surgical Margins of Oral Squamous Cell Carcinoma Using Frozen Sections: A Practical Clinicopathological Management for Recurrences

**DOI:** 10.1155/2014/823968

**Published:** 2014-06-24

**Authors:** Shun Miyota, Takanori Kobayashi, Tatsuya Abé, Hisashi Miyajima, Masaki Nagata, Hideyuki Hoshina, Tadaharu Kobayashi, Ritsuo Takagi, Takashi Saku

**Affiliations:** ^1^Division of Oral and Maxillofacial Surgery, Niigata University Graduate School of Medical and Dental Sciences, Niigata 951-8514, Japan; ^2^Department of Dentistry and Oral Surgery, Aizu Chuo Hospital, Aizu Wakamatsu 965-8611, Japan; ^3^Oral Pathology Section, Department of Surgical Pathology, Niigata University Hospital, Niigata 951-8520, Japan; ^4^Division of Oral Pathology, Niigata University Graduate School of Medical and Dental Sciences, 2-5274 Gakkocho-dori, Chuo-ku, Niigata 951-8514, Japan; ^5^Oral Implant Clinic, Niigata University Hospital, Niigata 951-8520, Japan; ^6^Division of Reconstructive Surgery for Oral and Maxillofacial Region, Niigata University Graduate School of Medical and Dental Sciences, Niigata 951-8514, Japan

## Abstract

*Background*. Local recurrence remains a challenging clinical issue for the treatment of oral squamous cell carcinoma (SCC). We analyzed retrospectively how effective the frozen section technique (FS) was against recurrences of oral SCC. *Methods*. We screened 343 surgical samples from 236 patients who had oral SCC, carcinoma in situ (CIS), or epithelial dysplasia, and we followed up their clinical outcomes for at least 5 years. Histopathological states of surgical margins were compared between FS and surgical materials in relapse and relapse-free groups, respectively. *Results*. Among the 236 patients, 191 were classified into the relapse-free group, and 45 into the relapse group. FS was more frequently performed in the relapse-free group (128/191) than in the relapse group (83/152). Histopathologically, moderate dysplasia or CIS (borderline malignancies) and SCC were recognized in 55 samples of the relapse-free group and in 57 of the relapse group. For those surgical margins with borderline malignancies, additional incisions were performed in 38 of the 55 relapse-free cases, which reduced to 20 from the 38 margins with borderline malignancies (47.4% reduction), and in 39 of the 57 relapse cases, which reduced to only 3 of 39 (7.7% reduction). *Conclusions*. The intraoperative assessment of surgical margins by FS is essential in preventing recurrences of oral mucosal malignancies.

## 1. Introduction

The incidence of oral cancers as well as the mortality from oral cancers, most of which are histopathologically squamous cell carcinoma (SCC), has been increasing in the last six decades in Japan as well as in European and Asian countries [1–3]. In south Asian countries especially, oral cancer is one of the most common forms of cancer [4–6], and in recent years the control and prevention of oral cancer have been receiving increased attention from the international oral health care community [7]. Among the major three therapeutic strategies—surgery, irradiation, and chemotherapy—the first choice for oral cancer is still surgery, even though various anticancer agents, including molecular target drugs, are now available [8].

The recent increase in the incidence of all types of cancer, including oral cancer, in Japan is partly due to longevity and improved detection of early stage lesions [9]. In addition to the two background factors mentioned above, there is one special situation for oral cancer: superficially spread varieties, which we have called superficial carcinoma, have recently been increasing in number [3, 10–12]. Different from the classic phenotype of oral cancer characterized by exophytic growth with intrinsic invasion and ulceration [13], superficial carcinoma is a lesional complex of different borderline lesions including epithelial dysplasia, carcinoma in situ (CIS), and microinvasive SCC, and it requires a more critical setting of surgical margins than classic SCC because the judgment of malignant-lesional extension can be difficult for both surgeons and pathologists. To help resolve such a difficult situation, we have developed objective diagnostic concepts and practices for oral borderline malignancies [10–12, 14, 15].

To this end we have also distinguished two categories of oral SCC:* de novo* and sequential types. The former is a conventional type of SCC without precursor lesions in vicinity of the main cancer foci, while the latter is based on superficial types surrounded by precursor lesions. This sequential type of SCC, which accounts for approximately 60% of tongue SCCs, is characterized by its predominance in elderly women, who less frequently smoke cigarettes or drink alcohol than patients with* de novo *type SCCs [3].

The biggest issue in clinical interventions for superficial SCC has been postoperative local recurrences. To prevent local recurrences, we have recently introduced the frozen section technique (FS) to confirm surgical margins by the above-mentioned histopathological criteria which we developed over the last 10 years in our hospital. There have been extremely few reports which have carefully examined the effectiveness of FS in association with recurrence-based prognoses. Therefore, in this study, we analyze how effective FS has been against recurrences of oral SCC, especially for superficial types.

## 2. Materials and Methods

### 2.1. Patients and Preparation of Samples

We selected 236 cases of oral cancer with at least 5-year follow-up data and sufficient clinical data from 343 samples of oral cancer which had been diagnosed clinically as oral SCC or leukoplakia and had been documented in the surgical pathology files of the Division of Oral Pathology, Niigata University Graduate School of Medical and Dental Sciences, during a 6-year period from 2002 to 2007. In addition to such patient clinical data as age, sex, tumor location, and tumor size (T factors), we were able to ascertain history of local recurrence from clinical records of the 236 patients and determine intervals between the last surgeries and recurrences. Since samples of recurrent cases were included, the total number of samples (tissue specimens) screened was larger than the number of cases. According to their local recurrence histories, the samples were classified into relapse and relapse-free groups, respectively. These cases separated into the two groups did not metastasize to regional lymph nodes or distant organs. There were 152 relapse and 191 relapse-free samples, for a total of 343. Cases of SCC, CIS, and epithelial dysplasia (mild and moderate) which recurred after primary surgery were categorized into the relapse group, while those without recurrence for at least five years after surgery were categorized into the relapse-free group. The study protocol for analyzing clinical records and surgical specimens was reviewed and approved by the Ethical Board of the Niigata University Graduate School of Medical and Dental Sciences (Oral Life Science).

During surgical operations, FS samples were obtained by pathologists from surgical margins (anterior, posterior, upper, and lower margins of mucosal surfaces and bottom margins towards muscle layers) of* en bloc* removed specimens for oral cancer or leukoplakia, which included histopathological varieties of SCC, CIS, and epithelial dysplasia. Those oral mucosal samples were snap-frozen and cut using a Leica cryostat CM1850 (Leica Biosystems, Wetzlar, Germany), and frozen sections were stained with hematoxylin and eosin (HE). HE-stained frozen sections were crosschecked and diagnosed by at least two pathologists. When frozen sections were diagnosed as moderate dysplasia or more advanced lesions, pathologists recommended the additional removal of surgical margins.

The surgical materials were fixed routinely in 10% formalin and embedded in paraffin. Serial 3 *μ*m sections were cut from paraffin blocks. One set of the sections was stained with HE and the others were used for immunohistochemistry and for pathological diagnoses.

### 2.2. Intraoperative Assessment of Surgical Margins by FS

We calculated the rate of performing FS during surgery in both relapse and nonrelapse groups, respectively, and their histopathological diagnoses by FS during surgery were reconfirmed by their paraffin sections from the same specimens used for FS. During surgery, excisional margins were evaluated and assigned to one of the following categories: normal, epithelial hyperplasia, epithelial dysplasia (mild and moderate), CIS, or SCC. We regard moderate dysplasia as true dysplasia, one of the borderline malignancy categories in addition to CIS [10–12]. Therefore, when pathologists diagnosed a case as moderate dysplasia or worse, they immediately reported their findings to the operating room to allow for additional surgical removal.

Tissue samples obtained by additional incisions were also counted as samples, although they were diagnosed on paraffin sections only and not on frozen ones because we did not perform the second FS after additional excisions. Those histopathological diagnostic criteria were commonly shared by surgeons and pathologists, and reasons for additional surgery recommendations were mutually understood by them. Our diagnostic criteria for borderline malignancies including CIS and epithelial dysplasia were not always identical to those by WHO 2005 [16], but were already described elsewhere [10–12, 14, 15]. We regarded severe epithelial dysplasia according to the WHO standard as a synonym with CIS.

### 2.3. Postoperative Evaluation of Surgical Margins by Paraffin Sections

Surgical specimens were routinely fixed in formalin and embedded in paraffin, from which 5–7 mm-thick slices were frontally prepared from posterior to anterior directions. Sections were obtained from every tissue slice, stained with HE, and evaluated by three independent pathologists. In addition to histopathological diagnoses for the main foci of the surgical specimens, their surgical margins were carefully checked and evaluated into the same six categories—from normal epithelia, epithelial hyperplasia, epithelial dysplasia, mild and moderate, and CIS, to SCC—as mentioned above. In case additional surgeries were performed, additionally incised specimens were regarded as actual surgical margins. For histopathological diagnoses for both main foci and surgical margins, we performed immunohistochemistry to make objective judgments based on our diagnostic criteria for borderline malignancies as mentioned above.

When multiple and different foci were included in one specimen and thus multiple diagnoses were obtained, those of the most severe grades were regarded as final diagnoses of the main lesions. Those cases were further investigated for follow-up studies by referring to their final diagnoses for the surgical specimens. When categorized into the relapse group, we compared frozen sections and biopsy or surgical specimens from recurrent lesions to determine whether or not they could be regarded as recurrence and calculated the intervals from FS to surgery for recurrent lesions.

### 2.4. Statistical Analysis

Clinical data were statistically analyzed using GraphPad Instat (version 3.06 for Windows, GraphPad Software, San Diego, CA, USA). The average scores for patients' ages were calculated using a *t*-test, and the clinicopathological items, such as sex, location, local recurrence, and performance of FS, were studied using Fisher's exact test. *P* < 0.05 was considered statistically significant.

## 3. Results

### 3.1. Histopathology and Tumor Size of Primary Lesions

Among the 236 cases, 191 (80.9%) were classified into the relapse-free group and 45 (19.1%) into the relapse group. In the 191 cases of the relapse-free group, 120 (62.8%) were diagnosed as SCC, 44 (23.0%) as CIS, 11 (5.8%) as moderate epithelial dysplasia, and 16 (8.4%) as mild epithelial dysplasia. In the 45 cases of the relapse group, 35 (77.8%) were SCC, 6 (13.4%) CIS, 3 (6.7%) moderate epithelial dysplasia, and 1 (2.2%) mild epithelial dysplasia (Table 1).

In terms of SCC cases, we categorized 120 SCC cases of the relapse-free group and 35 of the relapse group according to their T factors (tumor size). In the relapse-free group, there were 30 T1 (15.7%), 41 T2 (21.5%), 8 T3 (4.1%), and 41 T4 (21.5%). The relapse group included 7 T1 (15.6%), 12 T2 (26.7%), 5 T3 (11.1%), and 11 T4 (24.4%). There were no statistically significant differences between T factors and relapse tendencies (*P* value: T1: *P* = 0.655; T2: *P* = 1.000; T3: *P* = 0.171; T4: *P* = 0.841), indicating that T factors did not affect relapsing of lesions (Table 1).

### 3.2. Age and Sex

Out of the 236 patients, 133 (56.4%) were males, and 103 (43.6%) were females, with an overall male-to-female ratio of 1.3 : 1. Their ages ranged from 21 to 92 years, and the average was 67.2 years (male 64.2, female 69.8, resp.).

Among the 191 relapse-free patients, there were 115 males (60.2%) and 76 females (39.8%) with a male-to-female ratio of 1.5 : 1. In contrast, among the 45 patients of the relapse group, females (27, 60.0%) were more predominant than males (18, 40.0%) (Table 1). The difference in the male-to-female ratios was statistically significant between the two groups (*P* < 0.05).

**Figure 1:**
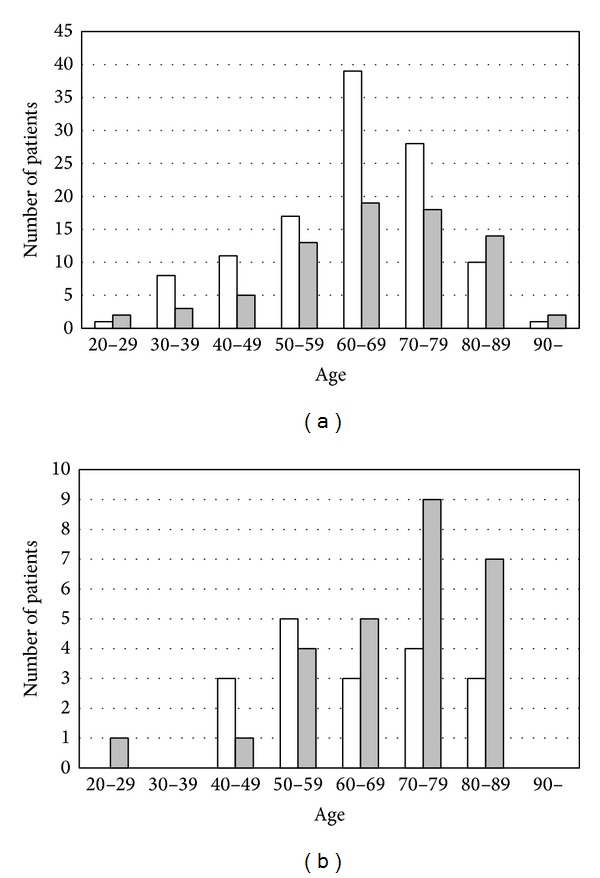
Age and sex distribution of patients with squamous cell carcinoma: relapse-free type (a) and relapse type (b). Open square: male; gray square: female. A total of 236 patients were analyzed in this study. There were 133 male (56.4%) and 103 female (43.6%) patients with an overall male-to-female ratio of 1.3 : 1. Their ages ranged from 21 to 92 years, and the average was 67.2 years (male 64.2, female 69.8, resp.). There were 191 relapse-free and 45 relapse patients. Among the 191 patients who were relapse-free, there were 115 males (60.2%) and 76 females (39.8%), with a male-to-female ratio of 1.5 : 1. In contrast, in the 45 cases with relapse, sex differences were reversed with 18 males (40.0%) and 27 females (60.0%). The mean age of the relapse-free group was 64.2 years (range from 21 to 92 years; male 63.0, female 65.9), while that of the relapse group was 67.2 years (range from 27 to 91 years; male 64.2, female 69.2). In the relapse patients, their sex difference was reversed as compared with the relapse-free group, and the difference was statistically significant (*P* = 0.019).

The age of the relapse-free group ranged from 21 to 92 years, with a mean of 64.2 years (63.0, males; 65.9, females), while that of the relapse group ranged from 27 to 91 years, with a mean of 67.2 years (males, 64.2; females, 69.2) (Figure 1). However, the difference in the mean ages was statistically not significant between the two groups, though the patients of the relapse group were definitely older on average than those of the relapse-free group.

### 3.3. Locations

In the relapse-free group, the most frequent sites were the tongue (77, 40.3%) and the gingiva (77, 40.3%) followed by the buccal mucosa (22, 11.5%) and floor of the mouth (15, 7.9%). However, in the relapse group, the gingiva was the most frequent site of lesions (22, 48.9%), followed by the tongue (14, 31.1%), buccal mucosa (7, 15.6%), and floor of the mouth (2, 4.4%). Statistically, there was no significant difference in site distributions between two groups (Table 1).

### 3.4. Intraoperative Assessment of Surgical Margins by FS

The intraoperative assessment by FSs was more frequently performed in the relapse-free group (128, 67.0%) than in the relapse group (83, 54.6%), with a statistically significant difference (*P* < 0.05) (Table 1).

Histopathological diagnoses of surgical margins by FS are shown in Table 2. Among the 128 samples of the relapse-free group, 8 margins (6.2%) were diagnosed as CIS, 47 (36.7%) as moderate epithelial dysplasia, and 61 (47.7%) as mild epithelial dysplasia. Borderline malignancies were found in 55 margins (43.0%). In contrast, in the relapse group, 5 margins (6.0%) were diagnosed as SCC, 24 (29.0%) as CIS, 28 (33.7%) as moderate epithelial dysplasia, and 20 (24.1%) as mild epithelial dysplasia among the total of 83 margins, demonstrating that true (SCC) and borderline (CIS and moderate dysplasia) malignancies (57, 68.7%) were more frequently recognized during surgery (*P* < 0.001, bold value sets in Table 2).

### 3.5. Additional Incisions according to FS

In the relapse-free group, among the 55 margins which contained borderline malignancies diagnosed by FS, 38 (69.1%) were additionally incised, while the remaining 17 margins were left behind because additional incisions were not surgically possible. In the relapse group, 39 (68.4%) among the 57 margins were additionally incised. There was no significant difference in the rates for additional surgery after FS (Table 3).

### 3.6. Total Histopathological Evaluation of Surgical Margins

To confirm whether the additional surgery according to FS was clinically useful, surgical margins were comparatively examined between paraffin sections of surgical specimens and frozen sections at surgery. Table 3 summarizes the final histopathological diagnoses of surgical margins, performances of FS, and additional surgery, which were compared between the relapse-free and relapse groups.

In the relapse-free group (*n* = 191), FS was performed in 128 cases but not in 63 cases. The 63 cases without FS, in which 8 margins with CIS and 33 margins with moderate dysplasia were included, did not relapse within the observation period of 5 years. Among the 128 cases, additional surgeries were recommended by pathologists in 55 cases because of the presence of borderline malignancies at their margins but not in 73 cases because there was no evidence of malignancy. Among the 55 cases, however, additional surgery was actually performed in only 38 cases (69.1%), while no additional surgery was performed for the remaining 17 cases (30.9%) due to such surgical reasons as limited mucosal spaces. Among the 38 cases, two cases were diagnosed as SCC (5.3%), 6 as CIS (15.8%), and 12 as moderate dysplasia (31.6%); these 20 cases with SCC and borderline malignancies (in total, 52.6%) at their surgical margins were free from recurrences. In the remaining 18 cases out of the 38 cases, their margins (47.3%) were diagnosed as mild dysplasia, which was considered to be due to additional surgeries.

In the relapse group (the lower part of Table 3), FS was performed in 83 samples (54.6%) but not in 69 cases (45.4%). More than half of them recurred even though FS was performed. Additional surgery was recommended for 57 (68.7%) but not for 26 cases (31.3%) of the 83 cases. Although borderline malignancies were not recognized in the 26 cases, 4 SCC, 6 CIS, and 8 moderate dysplasia were left behind at their surgical margins. Among the 57 samples with borderline malignancies, additional surgery was performed only in 39 cases (68.4%) but not in 18 cases (31.6%). Even though FS and additional surgeries were performed in those 39 cases, SCC was identified in 6 cases (15.4%), CIS in 17 (43.6%), and moderate dysplasia in 13 (33.3%), which accounted for 92.3% (only 7.7% of the cases were downgraded into mild dysplasia in final sections). In other words, additional surgeries were insufficient for removal of borderline malignancy lesions which were not confirmed by FS. Of the 18 samples without additional surgeries, SCC and borderline malignancies were identified in 100%. Among the 69 cases in which FS was not performed, 10 SCC, 32 CIS, and 13 moderate dysplasia were identified in their surgical margins (80.0%). Among the 26 samples which were determined to be absent for malignancies in FS, 18 samples (69.2%) were shown to have malignancies in final sections, which we refer to here as “false negatives.” This was because of the fact that FS did not always cover the entire surgical margin.

### 3.7. Prognostic Survey of Recurrent Cases

To investigate the risk of recurrences of the borderline malignancies, and to determine how long patients should be followed up for recurrences, all of the primary surgical margins of 84 lesions which recurred within 10 years were retrospectively reviewed. The results are summarized in Table 4.

Among the 84 surgical samples with recurrences, 35 were identified as CIS (41.7%), 22 as moderate dysplasia (26.2%), 16 as SCC (19.0%), 9 as mild dysplasia (10.7%), and 2 as normal or hyperplasia (2.4%). In terms of recurrent lesions, SCC was found in 50 samples (59.5%), CIS in 27 (32.1%), and moderate dysplasia in 5 (6.0%). Among the 50 recurrent SCC, 20 samples (40%) originated from CIS, and 12 each were from SCC and moderate dysplasia (24.0% each). Among the 16 surgical margins with SCC, 12 samples recurred as SCCs (75.0%) with an average interval of 22.5 months, and 3 as CIS (18.8%) in 12.3 months. Of the 35 surgical margins with CIS, 20 samples recurred as SCC (57.1%) in 25.1 months, and 15 as CIS (42.9%) in 14.1 months. Of the 22 surgical margins with moderate dysplasia, 12 recurred as SCC (54.5%) in 33.3 months and 6 as CIS (27.3%) in 42.7 months. The results indicated that the lesions which we had diagnosed as borderline malignancies (moderate dysplasia and CIS) possessed the potential to develop into SCC, and that it was necessary for any categories of oral epithelial lesions, including mild dysplasia, to be followed up for at least 4 years for possible recurrences.

## 4. Discussion

In this study, we demonstrated the importance of the intraoperative FS for surgical margins of oral SCC in the prevention of local recurrences. In our series of oral SCC cases which were surgically treated, additional surgery according to FS was shown to be effective at reducing recurrences from surgical margins, though FS was not applied to all of the cases. There have been several reports investigating the accuracy of FS [17–19]. However, there are only a limited number of concrete reports regarding the prognosis including recurrences after FS. The present study has provided for the first time data to show that FS should be an integral part of any surgical intervention for oral SCC. In addition, it is now obvious from the present prognostic survey of surgical margins that our histopathological diagnostic criteria function in predicting local recurrences from surgical margins.

It has been emphasized that the control of metastasis is the ultimate, most important issue in cancer treatment [20]. In the case of oral cancer, however, local recurrence is often a more basic and important issue to be solved because many of oral cancers arise in the background of field cancerization [21]. For instance, superficial carcinoma, a lesional complex, consists of CIS as a main focus, with scattered SCC foci in advanced forms, together with surrounding epithelial dysplasia [10]. Hence, it is not always easy for a surgeon to determine precise surgical margins with the naked eye during surgery. As objective aids for setting surgical margins, Lugol's iodine [22] or toluidine blue [23] solutions have been utilized to visualize mucosal areas containing malignant lesions. However, the effectiveness of these vital staining methods remains inconclusive because their staining modes have not been well correlated to histopathological criteria of borderline malignancies [22]. In addition, we have experienced difficulties in judging unstable staining results in our surgery. More recently, narrow-band imaging (NBI) has been introduced for detection of extension areas of oral cancers, especially those containing superficial carcinoma, to determine surgical fields [24]. The usefulness of NBI, which detects hemoglobin-derived blue rays, has been theoretically supported by our concept that intraepithelial blood vessels are characteristic to oral borderline malignancy lesions [25].

These surgical aids provide a reference only, whereas FS allows pathologists to evaluate surgical margins directly and histopathologically. There is no room for macroscopic determination of lesional extensions by individual surgeons, who must rely on past experience. As shown in the present study, oral SCC cases in which FS was performed showed significantly better prognoses.

We could not confirm the reasons why FS was not performed in the relapse group, but it was speculated that surgeons were unable to use FS when lesions were too small or when surgery was done under local anesthesia. We did not investigate the size factor of other lesions than SCC because our series were composed of various kinds of malignancies ranging from simple leukoplakia types to bulky and invasive SCC. However, independent of lesion size, it is obviously helpful for surgeons to receive detailed histopathological reports directly concerning their own surgeries, which may further give feedback to their macroscopic judgments to improve treatment outcomes.

As shown in the present study, there were two major problems for FS as a tool for prevention of local recurrence to be solved. The first one was a matter of where to sample frozen sections. FS is applicable to only the marginal areas from which frozen sections are prepared but not to other areas which are not sampled for FS. Thus, inappropriate sampling can result in what we call “false negatives.” It is actually impossible to examine the whole surgical margin around a lesion; therefore only several representative points, such as front, rear, upper, and lower, of margins were selected for FS in our series. In case marginal areas are not examined by FS, there may be recurrences from those areas. To solve this problem, surgeons have to be experienced at judging where to check by FS. The second issue is insufficient extension of incisional areas in additional surgery. Surgical margins by additional incisions were not examined by FS in the present series, and it was not confirmed whether those extended margins were free from malignancies. Therefore, additional surgeries should be performed with enough extensive distance from borderline malignancy margins; otherwise, extended margins should be reexamined by FS. In reality, it is difficult to perform multiple FSs for one surgery because surgery time should be kept as short as possible to reduce the physical burden on patients. Thus, there is a need for surgeons and pathologists to discuss how to manage FS more effectively.

The present study has demonstrated a clear difference in clinical features between the relapse and relapse-free groups. Local recurrences were significantly frequent among elderly females whether the samples belonged to the relapse or relapse-free groups. The most characteristic feature in the relapse group samples was the high frequency of borderline malignancies, especially CIS, in their margins. As shown in Table 1, tumor sizes did not affect recurrences. In other words, whether SCCs recur or not depends on what is left behind in the surgical margins but not on T factors. This was also the most characteristic feature of oral superficial carcinoma, which was characterized as a model of field cancerization some 60 years ago [21]. According to our clinicopathological survey of patients with tongue SCC [3], 63% of them were classified as having sequential type, while 37% had* de novo* types. The sequential types tend to recur and occur in multiple sites. Regarding precursor lesions to oral SCC, we have proposed a new disease entity, orthokeratotic dysplasia [26, 27], which tends to arise next to SCC foci. From the pathologist's point of view, it is obvious that what we defined as borderline malignancies—including moderate dysplasia with two-phase appearances and orthokeratotic dysplasia, as well as varieties of CIS—was valuable as histopathological criteria for FS.

According to the findings of the present survey for recurrences, patients should be followed up for at least 4 years even if their surgical margins are free from borderline malignancies. In cases where borderline malignancy lesions were left behind, their outcome for recurrence intervals could be predicted from 12 to 47 months dependent on malignant grades from moderate dysplasia, CIS, and SCC. One of the important lessons from the present study was that any of the oral borderline malignancy categories possesses a potential to progress into SCC.

## 5. Conclusions

It should be noted that most oral mucosal lesions have risks of recurrence from their surgical margins and that the intraoperative assessment of margins using FS was effective in preventing recurrences of oral mucosal malignancies. In order to improve surgical treatment outcomes, surgeons and pathologists should share a common viewpoint on surgical margins.

## Figures and Tables

**Table 1:** Clinicopathological summary and recurrence status of 236 cases of oral epithelial lesions.

Clinicopathological parameters	Clinical outcomes				*P*-value for difference between relapse-free and relapse groups
	Relapse-free (*n* = 191)		Relapse (*n* = 45)	
	Number of cases	Ratio (%)	Number of cases	Ratio (%)
Sex					
Male	115	60.2	18	40.0	0.019
Female	76	39.8	27	60.0	

Location					
Tongue	77	40.3	14	31.1	0.308
Gingiva	77	40.3	22	48.9	0.317
Buccal mucosa	22	11.5	7	15.6	0.454
Floor of the mouth	15	7.9	2	4.4	0.537

Histopathological diagnosis of main focus					
Squamous cell carcinoma (SCC)	120	62.8	35	77.8	0.080
T1	*30 *	*15.7 *	*7 *	*15.6 *	0.655
T2	*41 *	*21.5 *	*12 *	*26.7 *	1.000
T3	*8 *	*4.1 *	*5 *	*11.1 *	0.171
T4	*41 *	*21.5 *	*11 *	*24.4 *	0.841
Carcinoma in situ (CIS)	44	23	6	13.4	0.223
Epithelial dysplasia, moderate	11	5.8	2	4.4	1.000
Epithelial dysplasia, mild	16	8.4	2	4.4	0.538

Intraoperative assessment of surgical margins by frozen sections					
Yes	128	67.0	83	54.6	0.025
No	63	33.0	69	45.4	

**Table 2:** Clinical outcomes and histopathological diagnoses of surgical margins by frozen section techniques (FS).

Clinical outcomes	Histopathology of surgical margins by FS											
	SCC	(%)	CIS	(%)	Epithelial dysplasia				Others	(%)	Total	(%)
					Moderate	(%)	Mild	(%)				
Relapse-free	0	(0.0)	8	(6.2)	47	(36.7)	61	(47.7)	12	(9.4)	128	(100.0)
Relapse	**5**	**(6.0)**	**24**	**(29.0)**	**28**	**(33.7)**	20	(24.1)	6	(7.2)	83	(100.0)

Total	5	(2.4)	32	(15.2)	75	(35.5)	81	(38.4)	18	(8.5)	211	(100.0)

*(**bold**) Differences between the relapse-free and relapse group were statistically significant (*P* < 0.001) in three categories of oral malignancies.

**Table 3:** Additional surgery according to FS and final histopathology at margins of surgical specimens.

Clinical outcomes	FS		(%)	Borderline malignancies recognized by FS		(%)	Additional Surgery according to FS		(%)	Final histopathological diagnoses at surgical margin									
										SCC	(%)	CIS	(%)	Epithelial dysplasia				Normal	(%)
														Moderate	(%)	Mild	(%)		
Relapse-free (*n* = 191)	Yes	128	(67.0)	Yes	55	(43.0)	Yes	38	(69.1)	2	(5.3)	6	(15.8)	12	(31.6)	18	(47.3)	0	(0.0)
							No	17	(30.9)	0	(0.0)	3	(17.6)	8	(47.1)	5	(29.4)	1	(5.9)
				No	73	(57.0)	—	—	—	1	(1.4)	1	(1.4)	11	(15.0)	57	(78.1)	3	(4.1)
	No	63	(33.0)	—	—	—	—	—	—	0	(0.0)	8	(12.7)	33	(52.4)	22	(34.9)	0	(0.0)

Relapse (*n* = 152)	yes	83	(54.6)	Yes	57	(68.7)	Yes	39	(68.4)	6	(15.4)	17	(43.6)	13	(33.3)	3	(7.7)	0	(0.0)
							No	18	(31.6)	3	(16.7)	9	(50.0)	6	(33.3)	0	(0.0)	0	(0.0)
				No	26	(31.3)	—	—	—	4	(15.4)	6	(23.1)	8	(30.8)	7	(26.9)	1	(3.8)
	No	69	(45.4)	—	—	—	—	—	—	10	(14.5)	32	(46.4)	13	(18.8)	13	(18.8)	1	(1.5)

**Table 4:** Histopathology of surgical margins where malignant lesions recurred.

Histopathological diagnosis of margins by previous surgeries	Numbers (%) of recurrent lesions and intervals (month) until recurrences														
	Epithelial dyspl asia							CIS			SCC			Total	
	Mild			Moderate								
	Number	(%)	Period	Number	(%)	Period	Number	(%)	Period	Number	(%)	Period	Number	(%)	Period
Normal/hyperplastic epithelia	0	(0.0)	0.0	0	(0.0)	0.0	1	(50.0)	12.0	1	(50.0)	43.0	2	(100.0)	27.5
Epithelial dysplasia	1	(3.2)	11.0	5	(16.1)	28.4	8	(25.8)	41.8	17	(54.9)	26.1	31	(100.0)	30.0
Mild	*0 *	(*0.0*)	*0.0 *	*2 *	(*22.2*)	*18.5 *	*2 *	(*22.2*)	*39.0 *	*5 *	(*55.6*)	*8.8 *	*9 *	(*100.0*)	*17.7 *
Moderate	*1 *	(*4.6*)	*11.0 *	*3 *	(*13.6*)	*35.0 *	*6 *	(*27.3*)	*42.7 *	*12 *	(*54.5*)	*33.3 *	*22 *	(*100.0*)	*35.0 *
CIS	0	(0.0)	0.0	0	(0.0)	0.0	15	(42.9)	14.1	20	(57.1)	25.1	35	(100.0)	20.4
SCC	1	(6.2)	2.0	0	(0.0)	0.0	3	(18.8)	12.3	12	(75.0)	22.5	16	(100.0)	19.3

Total	2	(2.4)	6.5	5	(6.0)	28.4	27	(32.1)	22.0	50	(59.5)	25.1	84	(100.0)	23.9
